# Does CEO tenure moderate the link between corporate social responsibility and business performance in small- and medium-sized enterprises?

**DOI:** 10.3389/fpsyg.2022.1037245

**Published:** 2022-12-06

**Authors:** Sae-Mi Lee, Paresha N. Sinha, Jee-Eun Bae, Yong-Ki Lee

**Affiliations:** ^1^The Center for Regional Development, Chonnam National University, Gwangju, South Korea; ^2^School of Management and Marketing, University of Waikato, Hamilton, New Zealand; ^3^The Department of AI Convergence, Hoseo University, Asan, South Korea; ^4^School of Business, Sejong Sustainability Energy Environment Bio Institute, Sejong University, Seoul, South Korea

**Keywords:** corporate social responsibility, business performance, small- and medium-sized enterprises, CEO tenure, echelons theory, stakeholder theory

## Abstract

This study investigates the effect of CSR activities on business performance of small- and medium-sized enterprises (SMEs) in South Korea setting. Based on upper echelons theory and stakeholder theory, the study further examines CEO tenure as a potential moderator between CSR activities and business performance. The study considers four dimensions of CSR (economic, legal, ethical, and philanthropic) and two types of business performance (financial and non-financial). To test the moderating effect of CEO tenure, we divided the sample into two groups: companies with short-term tenured CEOs and long-term tenured CEOs. The data were collected from 443 CEOs of SMEs in South Korea. We used a multi-group analysis with SmartPLS 4. The study finds that CEO tenure moderates the relationship between dimensions of CSR and business performance. More specifically, the study finds that CEOs in early-stage tenure focus on philanthropic activities to drive financial performance, while their counterparts focus on economic/legal dimension. CEOs, regardless of the length of tenure, consider the philanthropic dimension helpful for improving both financial and non-financial performance. This study expands prior research by examining the relationship between CSR and business performance in SMEs, considering the impact of the CEO tenure. The findings of this study make contributions to the literature by demonstrating that CEO tenure is an important factor in linking CSR to business performance. This research also adds evidence to the CSR literature that economic and legal dimensions are considered mandatory responsibilities, and CEOs of SMEs view them as interconnected. For practical implications, this study identifies different predictors of financial performance for companies with short-term vs. long-term CEO tenure. Short-term CEOs focus on philanthropy to improve financial performance, and both long- and short-term CEOs believe that philanthropy affects the company’s financial and non-financial performance.

## Introduction

The society’s increasing demand for companies being socially responsible have pressured companies to consider corporate social responsibility (CSR) strategies (Ahn and Park, 2018). Companies are expected to engage in CSR activities to meet the expectations of various stakeholders, while maximizing profits (Jenkins, 2009; Russo and Perrini, 2010; Lee et al., 2012). However, while CSR has become an important strategic initiative even for small- and medium-sized enterprises (SMEs; Graafland et al., 2003; Russo and Perrini, 2010; Stoian and Gilman, 2017), SMEs’ lack of knowledge of regulations, corruption, and lack of awareness of the benefits of CSR remain impediments to the implementation of CSR (Ciliberti et al., 2008). SMEs, unlike large firms, rarely have formal CSR strategies or budget allocation in place. If they engage in CSR activities, they do so at random. CEOs of SMEs are primarily responsible for making CSR-related decisions (Thornton and Byrd, 2013). The philanthropic dimension of CSR is important for understanding the CEO’s motivation and informal nature of CSR in SMEs (Jamali et al., 2009; Cheffi et al., 2021). CSR activities in SMEs may take different forms including providing jobs to the socially disadvantaged, investing part of corporate profits to social causes, and making donations to charities. Implementation of CSR can help improve businesses image (Pastrana and Sriramesh, 2014) and attract talented employees to achieve innovation outcomes (Bocquet et al., 2019) However, the cost associated with CSR is a challenge for SMEs who have limited financial resources and time. This is one of the reasons why SMEs are behind large firms in addressing environmental issues (Shashi et al., 2018).

Because companies exercising CSR are perceived as good citizens, various stakeholders including employees, customers, consumer activists, and local communities tend to have positive attitudes toward them. For example, employee job satisfaction and customer loyalty increase when SMEs are involved with CSR activities (Pivato et al., 2008; Jenkins, 2009; Lee et al., 2012). Prior research also shows that a franchisor’s engagement in economic and philanthropic CSR activities positively influences the franchisees’ economic and social satisfaction with the franchisor and loyalty (Hur et al., 2019). CSR activities related to the workforce have been found to be useful for avoiding sales decline. However, environment-related CSR activities were found to negatively impact SMEs’ growth (Stoian and Gilman, 2017). This suggests that prior studies on the effect of CSR activities may be mixed or inconclusive. For example, some studies report external pressures do not play a significant moderating role between the antecedents of CSR and CSR practices (Cheffi et al., 2021), while others show stakeholder salience and proximity play an important moderating role between CSR and financial performance in SMEs (Magrizos et al., 2021). Such mixed results have encouraged researchers to question validity or effectiveness of the CSR strategies for SMEs (Stoian and Gilman, 2017). Chen et al. (2019) and Khan et al. (2020) confirmed that CEO tenure had a negative effect on non-financial performance (CSR performance, corporate social and environmental performance). This result alludes to the importance of CSR for SMEs (Han and Park, 2018; Kang et al., 2021). It is possible that CEOs with different career horizons focus on different types of CSR activities. A finding that the key stakeholders were not confident about the CEO’s capabilities (Khan et al., 2020) during the early tenure suggests that CEOs may choose to prove themselves by focusing on risky CSR investments. This strategy may help them reap benefits in the future in the forms of higher compensation and business performance.

This study expands prior research by examining the relationship between CSR and business performance in SMEs, considering the impact of the CEO tenure. Some SME studies have examined the relationship between unidimensional CSR and unidimensional performance (Bahta et al., 2021; Cheffi et al., 2021; Palacios-Manzano et al., 2021) and investigated the roles of corporate reputation, corporate image, and business uncertainties as mediators or moderators. Other studies have used three dimensions including economic, social, and environmentally proactive CSR (Costa and Menichini, 2013; Torugsa et al., 2013) to discuss the importance of CSR. These studies omitted the philanthropic dimension, which is important for understanding the CEO’s motivation and informal nature of CSR in SMEs (Jamali et al., 2009; Cheffi et al., 2021). Studies that focused on the relationship between the CEO personal attributes (age, personality, and education level), CSR, and financial performance (Li et al., 2020) have not considered different types of performance or various dimensions of CSR. It seems important to consider multiple dimensions of CSR beyond the company’s economic motives in studying the effect of CSR (Kim et al., 2017). Thus, this study considers various dimensions of CSR in an effort to offer a comprehensive understanding of SMEs’ CSR strategies and activities. This study will fill the void in the literature by offering empirical evidence on the relationship between various dimensions of CSR and financial and non-financial business performance.

With these motivations in mind, this study uses four dimensions of CSR of Carroll (1991) to provide a complete picture of CSR to SME managers who want to implement CSR. The purpose of this study is to examine the significance of economic, legal, ethical, and philanthropic dimensions of CSR in their effect on financial and non-financial performance of SMEs. Using upper echelons theory (Hambrick and Mason, 1984) and stakeholder theory (Donaldson and Preston, 1995; Lee, 2011), the current study examines whether CEO tenure moderates the relationship between four dimensions of CSR (economic, legal, ethical, and philanthropic) and two types of business performance (financial and non-financial).

This study uses upper echelons theory, as we acknowledge CEOs of SMEs exert huge influences on strategic decisions including CSR. CEOs play a significant role in formulating CSR strategies, which can help increase the firm’s revenue (Mubeen et al., 2021). Their opinions, values, experiences, and attitude toward CSR play a very important role in determining the companies’ directions (Morgeson et al., 2013; Wei et al., 2018). Prior research reports mixed results on the relationship between CEO tenure and business performance (e.g., financial performance; Friede et al., 2015). A study by Henderson et al. (2006) found that CEO tenure and financial performance were directly proportional to CEO tenure in the stable food industry, but opposite results were found in the dynamic computer industry. Therefore, the current study treats CEO tenure as a potential moderator between CSR and business performance.

Differently stated, this study attempts to understand whether the effects of CSR dimensions on business performance are different based on the CEO tenure. To test the hypotheses, the study uses a multi-group analysis with SmartPLS 4 on the data collected from South Korea. Korea was selected because a large portion of CSR activities were carried out by large firms, and not much was known about CSR activities of SMEs. The number of SMEs in Korea was about 7,286,000 as of 2020, an increase of about 989,000 compared to 2017. The number had been steadily increasing every year, and the number was 770 times more than that of large enterprises. SMEs accounted for about 99.87% of all Korean enterprises (Ministry of SMEs and Startups, 2022). Hence, it seemed necessary to focus on SMEs in South Korea to study the effects of CSR on business performance.

This study thus seeks to extend and reconcile prior CSR research by providing a more fine-grained depiction of different dimensions of CSR—CEO tenure—different types of performance relationships. The results will enrich literature by contributing in several ways. First, the study will show whether CEOs with short-term vs. long-term tenure use different CSR strategies and initiatives to enhance business performance. Thus, understanding how CSR strategies shift over time and how CSR activities impact the two types of performance will provide strategic guidance on the CSR initiatives and improve our understanding of CEO tenure as a potential moderator. Our study that explores CEO tenure as a moderator will also reveal the mechanism, through which CEO tenure exerts an influence on the relationship between CSR initiatives and financial and non-financial performance. This insight will be useful for evaluating SME’s competitiveness and strategies.

The second contribution is related to the finding about the relationship between different dimensions of CSR and business performance. The finding will show which dimension of CSR is utilized most by early- and late-stage CEOs in their efforts to improve the SMEs’ financial and non-financial performance. The result will link dimensions of CSR to different types of performance and offer some strategic implications to the CEOs.

## Literature review

### Corporate social responsibility in SMEs

Corporate social responsibility refers to the responsibility that a company should take as a member of a society beyond its economic responsibility of profit-seeking. The concept of CSR has evolved over the years, to include different types of CSR such as responsive, strategic (Porter and Kramer, 2006), implicit, and explicit CSR (Matten and Moon, 2008). While scholars in the earlier period defined CSR from the social purpose perspective (Bowen, 1953) or social responsiveness (Sethi, 1979), those in the later period embraced a complementary model. For example, Lantos (2001) offers three types of CSR: ethical, altruistic, and strategic responsibility.

**Figure 1:**
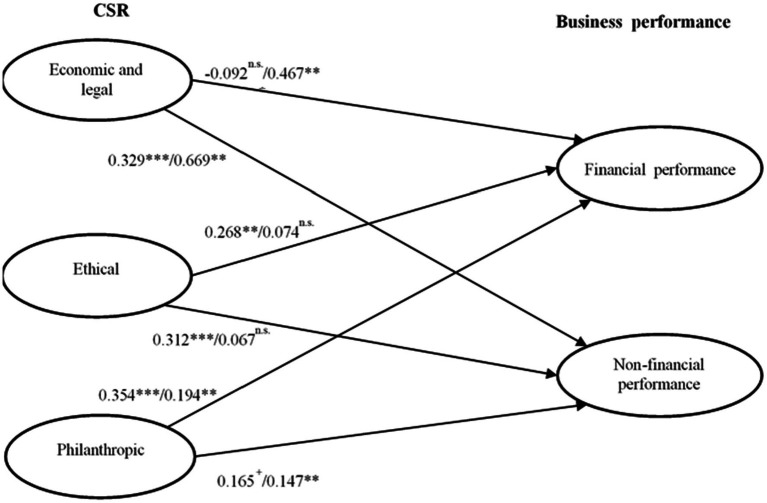
Estimates of structural model. Numbers: Short-term tenured CEOs/Long-term tenured CEO, ***p < 0.001, **p < 0.01, *p < 0.05, ^+^*p* < 0.1, n.s. = non significant.

However, model of Carroll (1991) comprising four dimensions of CSR addresses not only basic and mandatory corporate responsibilities but also social and voluntary responsibilities. Thus, this study uses four dimensions of CSR of Carroll (1991) (Figure 1). Economic responsibility, also known as the primary responsibility, is about generating profits from the business operation. Legal responsibility refers to the duty to comply with the rules and regulations that the business is subjected to. Both economic and legal responsibilities are considered basic mandatory requirements for the business to survive in the market. Ethical responsibility addresses a duty to meet the generally accepted ethical standards within the society. Philanthropic responsibility is concerned with making a contribution to the improvement of the community as a good corporate citizen. This responsibility is related to making discretionary and voluntary contributions to the society.

There is a vast amount of research that suggests CSR has become an important strategic initiative even for small- and medium-sized enterprises (SMEs; Graafland et al., 2003; Russo and Perrini, 2010; Stoian and Gilman, 2017) due to their close connection with the community and stakeholders. Unlike large firms that have resources (Fombrun, 1996), SMEs that lack financial resources may view adoption of environmentally-conscious business practices as an operational and financial risk (Jenkins, 2004). Hence, one stream of research suggests that SMEs will be less likely to implement CSR without regulatory pressures because of the lack of resources and perceived costs associated with the CSR implementation (Jenkins, 2004; Lee et al., 2016). Furthermore, external pressures (e.g., stakeholder pressures) were found to have no significant effect on CSR practices among SMEs (Cheffi et al., 2021). Another research stream that paid attention to the limited financial and business resources and managerial expertise (Magrizos et al., 2021) explains the heterogeneous implementation of CSR and absence of formal strategies and budget allocation. Despite these challenges, some SMEs have implemented CSR informally, driven by intrinsic values of the CEOs (Jenkins, 2004) to improve their businesses image (Pastrana and Sriramesh, 2014) and to attract talented employees who can help improve the firm’s innovation outcomes (Bocquet et al., 2019). Previous studies on Australian and Pakistan SMEs found the adoption of each CSR dimension was influenced by the company’s capabilities, and each CSR dimension affected financial performance differentially (Torugsa et al., 2013; Ikram et al., 2019).

### Characteristics of CEOs and CSR

Upper echelons theory (Hambrick and Mason, 1984) states that executives rely on their personal characteristics, such as experience, value, attitude toward CSR, and personality in making business-related decisions. The theory suggests that CEOs’ personal characteristics influence strategic decision-making and organizational performance (Hambrick and Mason, 1984; Carpenter et al., 2004). Based on the theory, this study views that CEOs’ characteristics will play a role in determining companies’ priorities and preferences related to CSR. Previous studies show that CEOs’ characteristics influence CSR activities and performance (Carpenter et al., 2004; Petrenko et al., 2016; McCarthy et al., 2017; Khan et al., 2020). Some studies that had examined CEO tenure as one of the CEO characteristics reported mixed results on the relationship between CEO tenure and CSR performance (Khan et al., 2020). However, more recent studies show that CEO tenure is related to CSR performance (Oh et al., 2018; Chen et al., 2019). The studies suggest that CEOs tend to take strategic risks in the early stage of their tenure. As they remain in the position for a long time, they run the company based on their own fixed paradigm and maintain the status quo without seeking changes (Hambrick and Fukutomi, 1991). In addition, Khan et al. (2020) show that social and environmental performance was higher for companies with short-term tenured CEOs than those with long-term tenured CEOs. In other words, they find an inverse relationship between CEOs’ tenure and companies’ focus on CSR. One possible interpretation of this finding is that short-term tenured CEOs may be more sensitive to demands and expectations from various stakeholders, and, thus, more willing to focus on CSR. On the other hand, long-term tenured CEOs are better equipped to manage pressure from the external stakeholders as they have gained executive power and social capital (Onali et al., 2016). Inconsistent with the study of Khan et al. (2020), the study of Oh et al. (2018) shows a negative relationship between CEO tenure and corporate social irresponsibility (CSIR). Their study suggests that long-term tenured CEOs tend to avoid activities that are socially irresponsible. Thus, the studies on the relationship between CEO tenure and CSR are not consistent.

We view that CEOs of SMEs exert more influences on strategic decision-making compared to those of large corporations (Tran and Adomako, 2021). This is because decisions are often made centrally in SMEs. Thus, CEOs of SMEs are primarily responsible for making CSR-related decisions (Thornton and Byrd, 2013). Based on prior research, we view that examining CEOs’ tenure in SMEs is helpful for understanding how CEO tenure affects strategic decisions related to CSR. Prior study (Tran and Adomako, 2021) suggests a moderating role of CEO tenure in the relationship between SME CEOs’ social capital (e.g., capability to integrate stakeholders) and corporate social performance. The study shows that social capital of long-term tenured CEOs has a stronger effect on stakeholder integration. This makes sense because long-term tenured CEOs will have a stronger relationship with their external stakeholders (e.g., shareholders) than their counterparts.

## Hypothesis development

### The effect of CSR on business performance

Two major opposed theories have been used to explain CSR: agency theory and stakeholder theory. Agency theory posits that executives of large corporations act as an agent and not as an owner, and, thus, seek interests misaligned with those of the owners (shareholders). CSR studies based on the agency theory point out to the negative aspects of CSR activities. They argue that CSR activities may increase agency costs and decrease corporate performance (Wright and Ferris, 1997; Friedman, 2007; Hasan, 2021). The scholars based on the agency theory believe that CSR activities are driven by self-interested executives who often sacrifice the interest of the shareholders in their efforts to improve images of the company and themselves (Friedman and Friedman, 1990). However, many empirical studies suggest that CSR activities do not negatively influence corporate performance. For example, Li et al. (2016) show that CSR activities have a positive effect on corporate value improvement. The study rejects the hypothesis that powerful CEOs will make more investments in CSR activities in an effort to build their own reputation, indicating agency theory is not appropriate for explaining CEOs’ motives to engage in CSR.

Stakeholder theory is helpful for understanding positive aspects of CSR. The theory acknowledges the interconnected relationship between a company and its stakeholders (e.g., shareholders, employees, and customers; Naseem et al., 2020; Rehman et al., 2020; Nirino et al., 2022). This theory suggests that CSR activities should be planned and implemented in consideration of the needs and expectations of all stakeholders including creditors, suppliers, employees, consumers, and local communities (Freeman, 1984). Some recent studies show a positive relationship between CSR and business-related outcomes (Claver-Cortés et al., 2020). For example, Hou et al. (2016) show CSR activities have a positive effect on financial and operational performance. Similarly, several studies (Choongo, 2017; Juarez, 2017; Nejati et al., 2017; Moneva-Abadía et al., 2019) show a positive effect of CSR on performance among SMEs. These results are understandable because corporate engagement in CSR will enhance the relationship with stakeholders by addressing their needs. This study based on the stakeholder theory proposes that all four dimensions of CSR will have a positive effect on both financial and non-financial performance. This study presents the following hypotheses.

Most of the studies on CSR of SMEs were conducted mainly in Europe (Oduro et al., 2021). More recently, some studies examined the relationship between CSR and firm performance of SMEs in Africa and Asia. For example, Bahta et al. (2021) investigated the mediating role of firm reputation in the relationship between CSR and firm performance among SMEs in Eritrea, East Africa. Le (2022) identified the mediating roles of corporate image, corporate reputation, and customer loyalty in the relationship between CSR and corporate performance in Vietnam.

*H1*: Four dimensions of CSR (economic, legal, ethical, and philanthropic) positively affect financial performance of SMEs.

*H2*: Four dimensions of CSR (economic, legal, ethical, and philanthropic) positively affect non-financial performance of SMEs.

### Moderating role of CEO tenure

Upper echelons theory assumes that CEO characteristics affect strategic decision-making and corporate performance, and studies based on this theory have been continuing. This theory has also made substantial contributions to studies related to CEOs with a focus on observable trait variables about CEOs (Wang et al., 2016; Bassyouny et al., 2020). This theory also emphasizes changes in CEO behavior during the CEO tenure (Hambrick and Fukutomi, 1991). CSR-related decision-making is a part of corporate strategic decision-making, and in most companies, CSR-related decisions are made and implemented top-down (Bhattacharya et al., 2008). In other words, corporate CSR decision-making is made by the CEO, and the direction of CSR decision-making changes according to the characteristics of the CEO. Therefore, examining the influence of CEO characteristics on corporate CSR decision-making and outcomes will lead to a deep understanding of the organization (Kim et al., 2018). In particular, the tenure of the CEOs who have the final decision-making authority and responsibility of the company will be one of the most important determinants of the company’s CSR activities.

CEO tenure is known to influence the company’s strategic decisions and outcomes (e.g., performance and invention; Miller, 1991; Wu et al., 2005). While many studies examined the effect of CEO characteristics (e.g., age and value) on corporate performance, less emphasis has been placed on the role of CEO tenure in affecting CSR activities (Carpenter et al., 2004; Petrenko et al., 2016; Chen et al., 2019). Previous studies have demonstrated that CEO tenure can have different effects on the company performance (Nguyen et al., 2018). Studies show that the content and the influence of the characteristics of the CEO on the behavior of the company vary depending on the CEO tenure (McClelland et al., 2012). Longer CEO tenure appears to be detrimental to firm performance (Miller, 1991). Top executive tenure has an inverse U-shaped link with an organization’s financial performance (Miller and Shamsie, 2001).

This study proposes that long-term vs. short-term tenured CEOs exhibit different tendencies and attitudes toward CSR. Our rationale is as follows. Short-term tenured CEOs may feel the pressure to prove their capabilities to improve business performance and, thus, be more willing to take risks. In their efforts, they may focus on the higher levels of CSR activities because this can help companies set apart from the competitors (e.g., shoes companies donating a pair of shoes per sale to those in need). However, long-term tenured CEOs may not be interested in new or risky strategic initiatives. They may have already proven their capabilities to the shareholders, and do not feel the pressure to prove themselves. Thus, this study proposes that the effect of CSR dimensions on business performance (financial and non-financial) may differ based on the CEO tenure. This study offers the following hypothesis.

*H3*: The effect of four dimensions of CSR (economic, legal, ethical, and philanthropic) on business performance (financial and non-financial) will differ based on the CEO tenure. The effect of CSR on business performance will be greater for CEOs in the early stage than their counterparts.

## Materials and methods

### Sample and data collection

This study reached out to CEOs of SMEs that were registered with three different business associations in South Korea. The respondents were informed about the purpose of this study in person and requested to participate in the study. The surveyors who thoroughly understood the purpose of this study met with the CEOs of SMEs. Data collection lasted for 4 months. A pool of 1,100 respondents agreed to participate in the survey, and they received a copy of the questionnaire. In order to increase the response rate, participants who completed the questionnaire were offered a small gift. A total of 480 respondents completed the questionnaire. Forty-seven responses were discarded due to omission of important information. The total number of responses used for analysis was 433, leading to a response rate of 39.4% (433/1,100).

The demographic characteristics of the respondents are as follows (see Table 1). A significant number of respondents were males (81%). The sample was divided into two groups: short-term tenured CEOs (less than 3 years), referred to as Group 1, and long-term tenured CEOs (4 years and longer), referred to as Group 2. A majority of the short-term tenured CEOs (84%) worked for companies that had exited less than 4 years. On the other hand, more than half of the long-term tenured CEOs (61%) worked for companies that had existed for 11 years and longer. The difference between the two groups exists in sales. Almost all companies in Group 1 (90%) had annual sales of less than $500,000. About 59% of the companies in Group 2 had a minimum of annual sales of $4,250,000.

**Table 1:** Demographic profiles.

Category		Short-term tenure CEOs (G1) (*n* = 146)	Long-term tenure CEOs (G2) (*n* = 297)	Total (*n* = 443)
Gender	Male	108	251	359
	Female	38	46	84
Age	20–29	56	14	70
	30–39	69	48	117
	40–49	14	121	135
	50 and above	7	114	121
Business history (year)	1–3	123	15	138
	4–7	11	52	63
	8–10	2	50	52
	11–15	6	114	120
	Above 15	4	66	70
Number of employees	1–5	107	43	150
	6–10	27	37	64
	11–50	7	42	49
	51–100	2	95	97
	Above 100	3	80	83
Sales (Hundred million, KRW)	1–5	132	53	185
	6–10	7	42	49
	11–50	2	27	29
	51–100	2	97	99
	Above 100	3	78	81
Category	IT(Software)	55	94	149
	IT(Hardware)	14	46	60
	Manufacturing	36	46	82
	Distribution	8	21	29
	Service	33	58	91
	Law firm	-	30	30
	Franchise	-	2	2

### Common method bias assessment

Several procedural and statistical methods were used to reduce common method bias (Kang et al., 2021). One of the procedural methods involved explaining the purpose of the study to the respondents to increase the response rate (Podsakoff et al., 2003, 2012). Another procedural approach was concerned with modifying the questionnaire based on a pre-test result. The items that were confusing or hard to understand were removed after the pre-test. We also changed the order of questions on the questionnaire so that independent and dependent variables were not presented consecutively. In terms of the statistical approach, we ensured that VIF values were lower than the threshold value of 3.3 (Kock, 2015). All these test results support that common method bias was not a problem.

### Measures

We used a seven-point scale anchored by “1 = Strongly Disagree,” and “7 = Strongly Agree” to measure items. CSR dimensions in this study are defined as follows (Schwartz and Carroll, 2003; Lu et al., 2020). Economic and legal responsibility was defined as the extent to which a company engaged in socially responsible activities within the legal framework in pursuit of economic benefits for consumers and businesses. Twelve items were used to measure economic and legal CSR dimensions based on the previous studies (Maignan et al., 1999). Ethical responsibility was defined as a company’s ethically desirable responsibilities, both internally and externally. Philanthropic CSR was defined as responsibility in terms of social contribution taking into account employees and the community. Ethical and philanthropic responsibilities were measured with six items each. Both financial performance and non-financial performance were measured using five items each to assess business performance. While financial performance addressed the company’s quantifiable financial measures (e.g., sales, profits, and return on investment); non-financial performance was concerned with satisfaction judgment and brand perceptions of the stakeholders (e.g., customer satisfaction and employee satisfaction). Business performance items were adapted from the study of Banker et al. (2000).

## Results

### Measurement model

Convergent and discriminant validities of the measurement model were examined using SmartPLS 4.0.7.6. The result shows no evidence of discriminant validity between economic and legal CSR constructs. Thus, economic and legal dimensions were combined for further analyses. Several items that poorly performed during the measurement model assessment were removed for a purification purpose (see Table 2).

**Table 2:** Measurement model.

Constructs and items	Short-term tenured CEOs (*n* = 146)			Long-term tenured CEOs (*n* = 297)		
	Cronbach’s α, Standardized factor loadings	CR	AVE	Cronbach’s α, Standardized factor loadings	CR	AVE
**Economic and legal responsibility (ELR)**	0.908	0.926	0.609	0.911	0.928	0.616
Our business has a procedure in place to respond to every customer complaint.	-			-		
We continually improve the quality of our products (services).	0.799			0.736		
We use customer satisfaction as an indicator of our business performance.	0.785			0.730		
We have been successful at maximizing our profits.	-			-		
We strive to lower our operating costs.	0.663			0.798		
Top management established long–term strategies for our business.	0.743			-		
Our product (service) meets legal standards.	0.825			0.812		
Our contractual obligations are always honored.	0.842			0.830		
We have programs that encourage the diversity of our workforce (in terms of age, gender or race).	0.789			0.852		
Our company seeks to comply with all laws regulating hiring and employee benefits.	0.786			0.792		
International policies prevent employees’ compensation and promotion.	-			-		
The managers of this organization try to comply with the law.	-			-		
**Ethical responsibility (ER)**	0.870	0.902	0.607	0.897	0.920	0.658
Members of our organization follow professional standards	0.729			0.777		
Top managers monitor the potential negative impact of our activities on our community.	0.864			0.823		
We are recognized as a trustworthy company.	0.837			0.865		
Fairness toward co-workers and business partners is an integral part of our employee evaluation process	0.706			0.854		
A confidential procedure is in place for employee to report any misconduct at work (such as stealing or sexual harassment).	0.739			0.707		
Our salesperson and employees are required to provide full and accurate information to all customers.	0.786			0.829		
**Philanthropic responsibility (PR)**	0.753	0.860	0.676	0.759	0.860	0.674
Our business supports local and cultural activities.	-			-		
Our business gives adequate contributions to charity.	0.685			0.685		
Through internal policies, employees can adjust their work and personal life.	-			-		
Our company is interested in improving the overall level of society.	-			-		
I do not give up work even if it is difficult.	0.892			0.880		
We encourage partnerships with local businesses and schools.	0.872			0.883		
**Financial performance (FP)**	0.954	0.964	0.845	0.927	0.945	0.775
Over the past three years, our sales have increased.	0.927			0.869		
Over the past three years, our net profit has increased.	0.941			0.893		
Over the past three years, our company’s current ratio has been on the rise.	0.923			0.901		
Over the past three years, our investment returns have been on the rise.	0.907			0.849		
Over the past three years, our market share has been on the rise.	0.896			0.891		
**Non-financial performance (NFP)**	0.926	0.944	0.773	0.917	0.938	0.750
Employees are satisfied with our company.	0.797			0.861		
Customers are satisfied with our company.	0.902			0.875		
The brand image of our company is good.	0.914			0.897		
The reputation of our company is good.	0.929			0.861		
Our products (services) are more competitive than our competitors are.	0.850			0.838		

The measurement invariance was checked using the three-step MICOM procedure suitable for PLS-SEM (Hair et al., 2016). The following three steps were used: (a) configurational invariance assessment, (b) compositional invariance assessment, and (c) the equality of composite mean values and variances. To address the first step, the following tests were conducted. Cronbach’s α and composite reliability values were examined, which were over 0.7 for both groups. This indicates internal consistency of the measurement model. Factor loadings and AVE values were 0.5 or higher for both groups (Fornell and Larcker, 1981; Hair et al., 2006), suggesting convergent validity. The square root of the correlation coefficient and AVE values were examined to check for discriminant validity. As shown in Table 3, the square root of the AVE values was higher than the correlation coefficients. All values related to heterotrait-monotrait (HTMT; Henseler et al., 2015) were below 0.9, suggesting discriminant validity between the constructs. All these results support the configural invariance related to the first step. The second step was addressed by running a permutation test 1,000 times to verify the compositional invariance (see Table 4). The result shows that the values of c were higher than those of c*_u_*, indicating measurement invariance. The compositional invariance of all composite variables was established. The result confirms that the data were suitable for a multi-group analysis (Schlägel and Sarstedt, 2016). For the multi-group analysis, path coefficients of the two groups were compared (Group 1 and Group 2).

**Table 3:** Fornell-Larcker Criterion/Heterotrait-Monotrait ratio (HTMT).

	Short-term tenured CEOs					Long-term tenured CEOs				
	1	2	3	4	5	1	2	3	4	5
1. ELR	0.781					0.785				
2. ER	0.625/0.700	0.779				0.366/0.385	0.811			
3. PR	0.426/0.493	0.518/0.633	0.822			0.440/0.501	0.239/0.299	0.821		
4. FP	0.227/0.242	0.394/0.420	0.453/0.544	0.919		0.580/0.627	0.292/0.302	0.417/0.490	0.880	
5. NFP	0.595/0.644	0.603/0.665	0.467/0.549	0.513/0.545	0.879	0.758/0.829	0.347/0.362	0.458/0.531	0.711/0.770	0.866
Mean	5.56	4.95	5.04	4.31	5.20	5.08	4.63	4.94	4.69	4.83
SD	0.97	1.13	1.32	1.52	1.13	0.92	1.01	1.04	1.14	0.98

The square root of the variance shared between the constructs and their measures (AVE).

**Table 4:** Compositional invariance assessment (Step 2).

Constructs	Original correlation (c)	Correlation Permutation Mean	5.00% (c_u_)	Permutation *p*-Values
ELR	1.000	0.999	0.999	0.794
ER	0.998	0.998	0.994	0.325
PR	0.998	0.997	0.988	0.501
FP	1.000	1.000	0.999	0.999
NFP	1.000	1.000	1.000	0.057

The model fit was assessed based on the following criteria (Hair et al., 2016). First, VIF values were examined, which were lower than 3.3, indicating no evidence of multicollinearity. Second, *R*^2^ values, which indicate explanatory power of the model, were higher than 10%. Third, all Stone–Geisser’s Q^2^ values were greater than 0, suggesting predictive validity. Finally, all Standardized Root Mean Squared Residual (SRMR) values were lower than 1, confirming discriminant validity.

### Test of hypotheses

To test the hypotheses, the sample was divided into two groups: CEOs with short-term tenure (≤ 3 years) and CEOs with long-term tenure (4 years ≥) using a PLS multi-group analysis (see Table 5). This was done because the invariance test result indicated that the data were suitable for a multi-group analysis. We examined significance of the path coefficients and compared the path coefficients between the two groups. The test result shows that the effects of CSR dimensions on financial and non-financial performance vary depending on the CEO tenure. For companies with short-term CEOs, philanthropic and ethical CSR activities had a significant effect on financial performance. However, economic/legal CSR dimension was not found to have any effect. All three dimensions were found significant in their effects on non-financial performance. The effect sizes shown in Table 5 indicated that economic/legal dimension was the major predictor of non-financial performance for companies with short-term CEOs.

**Table 5:** Structural estimates across groups (PLS).

Paths	Short-term tenured CEOs (G1)				Long-term tenured CEOs (G2)				Estimate-difference	
	Estimate	*t*		*f* ^2^	Estimate	*t*		*f* ^2^		
ELR → FP	−0.092	1.014	n.s.	0.007	0.467	6.012	***	0.255	−0.558	***
ER → FP	0.268	2.504	**	0.051	0.074	1.492	n.s.	0.008	0.194	*
PR → FP	0.354	3.723	***	0.118	0.194	3.241	***	0.048	0.159	n.s.
ELR → NFP	0.329	3.578	***	0.120	0.669	12.013	***	0.816	−0.338	***
ER → NFP	0.312	3.934	***	0.096	0.067	1.575	n.s.	0.009	0.253	***
PR → NFP	0.165	1.691	*	0.036	0.147	3.020	**	0.043	0.017	n.s.
										
	R^2^	Q^2^			R^2^	Q^2^				
FP	0.245	0.342			0.373	0.443				
NFP	0.461	0.201			0.597	0.285				
										
SRMR	0.096				0.079					
VIF	1.399–1.881				1.165–1.363					

ELR, economic and legal responsibility; ER, ethical responsibility; PR, philanthropic responsibility; FP, financial performance; and NFP, non-financial performance. ^***^p < 0.01, ^**^p < 0.05, ^*^p < 0.10, and ^n.s.^non-significant.

For companies with long-term tenured CEOs, economic/legal dimension was the most significant predictor of financial performance. While philanthropic dimension was significant in affecting financial performance, its effect size was very small compared to that of economic/legal dimension. Ethical dimension was not found to have any effect on financial performance or non-financial performance. Economic/legal and philanthropic dimensions were found to have a significant influence on non-financial performance. These findings suggest that CEOs’ utilization of CSR strategies for improving the company’s financial and non-financial performance differs based on the tenure. Therefore, H1 (ELR → FP: β = −0.092, *p* = n.s./β = 0.467, *p* < 0.001, ER → FP: β = 0.268, *p* < 0.05/β = 0.074, *p* = n.s., and PR → FP: β = 0.354, *p* < 0.001/β = 0.194, *p* < 0.01) and H2 (ELR → NFP: β = 0.329, *p* < 0.001/β = 0.669, *p* < 0.001, ER → NFP: β = 0.312, *p* < 0.001/β = 0.067, *p* = n.s., and PR → NFP: β = 0.165, *p* < 0.1/β = 0.147, *p* < 0.01) that addressed the effects of dimensions of CSR on financial and non-financial performance were partially supported. H3 involving the moderating role of CEO tenure was partially supported (ELR → FP: β = −0.558, *p* < 0.001, ER → FP: β = 0.194, *p* < 0.1, PR → FP: β = 0.159, *p* = n.s., ELR → NFP: β = −0.338, *p* < 0.01, ER → NFP: β = 0.253, *p* < 0.01, and ER → NFP: β = 0.017, *p* = n.s.).

## Discussion of the findings

The purpose of this research was to examine the relationship between CSR and business performance, and test whether CEO tenure moderates the relationship between CSR and business performance. The study finding shows that short-term vs. long-term tenured CEOs weigh CSR dimensions differently in their strategies to improve business performance. For example, long-term tenured CEOs rely on the economic/legal dimension of CSR in their efforts to improve the company’s financial and non-financial performance. Short-term tenured CEOs, however, depend on the philanthropic dimension (not economic/legal dimension) to drive the company’s financial performance. The study also finds that long-term tenured CEOs do not consider the ethical dimension important for improving business performance. This viewpoint is different from their counterparts who view ethical CSR activities as important for improving both financial and non-financial performance. These findings are consistent with prior research (Oh et al., 2018; Khan et al., 2021).

This study offers four plausible explanations about the differences among CEOs in early- vs. late-stage tenure. The first one is related to their self-perceptions in terms of competencies, experiences, social capital, and capabilities to integrate stakeholders. Prior research suggests that short-term tenured CEOs lack stakeholder integration capabilities or social capital compared to their counterparts (Tran and Adomako, 2021). Thus, they may focus on addressing issues raised by external stakeholders (e.g., making contributions to the society) in an effort to meet their expectations. On the other hand, CEOs with long-term tenure may settle for their own paradigm (Hambrick and Fukutomi, 1991) and shy away from addressing the higher levels of CSR dimensions (e.g., philanthropic and ethical). This may happen because focusing on higher levels of CSR may take away their focus from maximizing profits.

The second explanation is related to CEOs’ use of CSR as evidence of corporate performance and a way of signaling about their capabilities. For example, Khan et al. (2021) report that CEOs in the early years of their tenure spend more efforts on corporate social and environmental activities, to decrease career-related concerns. Many companies are pressured by various stakeholders to engage in CSR activities. CEOs in early tenure may feel the pressure that they must engage in social responsibility (e.g., philanthropic) in order to prove their capabilities. Hence, they may use CSR activities to attract talented employees to improve innovation outcomes (Bocquet et al., 2019). On the other hand, CEOs who have been in the position for a long time may not feel the same level of pressure to prove themselves.

The third explanation is based on our speculation that CEOs’ motives behind CSR are self-interest driven. This viewpoint is consistent with prior research suggesting CEOs’ self-perception and narcissism influence the company’s strategies. Stock grant is a popular compensation tool used to incentivize and retain executives. Agency theory suggests that companies offer stock shares to CEOs in order to align their interests with owners’ (shareholders’). Long-term tenured CEOs tend to have a higher level of stake in the company because of the longer tenure. Thus, they may pursue activities such as economic activities (e.g., reducing operation cost and product development) that are directly related to the company’s financial performance. Improved financial performance will help stock price go up, which will positively affect CEOs’ personal economic gains. On the other hand, short-term tenured CEOs may not have the same level of stake in the company. Thus, their priority may not be to raise the company’s stock price in a short time, and they may be able to focus on other long-term initiatives (e.g., philanthropic activities) to reap rewards in their later years of service.

This study offers our fourth explanation based on the age gap between short-term tenured and long-term tenured CEOs. Many studies point out to the millennial generation’s strong interest in CSR. This study examined the age gap between the two CEO groups (Group 1 and Group 2) by using 40 years of age as the cutoff point in dividing the sample into two. About 86% of short-term tenured CEOs were under the age of 40 (millennial generation), while 21% of the long-term tenured CEOs were in that category. As expected, CEOs with short-term tenure are typically younger than their counterparts. We view that younger CEOs hold a much positive attitude toward CSR and are willing to seek philanthropic initiatives as one of the primary business strategies. This may be the reason why short-term tenured (young) CEOs view philanthropic CSR activities as the main driver of financial performance. Prior research supports our interpretation by showing CEO age moderates the relationship between CEOs’ tenure and CSR performance (Meier and Schier, 2021).

## Implications and future research

### Theoretical implications

This study finding adds evidence to the literature by showing that CEO tenure is an important factor in linking CSR to business performance. Based on upper echelons theory and stakeholder theory, this study examined the effects of four dimensions of CSR on business performance of companies with short-term vs. long-term tenured CEOs. The study shows that CEO tenure moderates the relationship between CSR dimensions and business performance. As CEOs stay in the position for a long time, they tend to lose sensitivity toward higher levels of CSR (e.g., philanthropic). CEOs in their early tenure are found to emphasize philanthropic activity as an important strategy for improving business performance. This finding indicates that CEOs in early vs. late tenure weigh low/high levels of CSR dimensions differently. While higher levels of CSR dimensions (ethical and philanthropic) are considered important by early-stage CEOs, lower levels of CSR dimensions (economic/legal) are viewed as drivers of business performance by late-stage CEOs. This study offered four explanations for the differences: (a) perceived capabilities to handle various stakeholders, (b) perceived pressure to prove themselves, (c) self-interest, and (d) age gap. Future studies may want to consider CEO tenure when they examine CSR strategies.

This study adds evidence to the CSR literature that economic and legal dimensions are considered mandatory responsibilities, and CEOs of SMEs view them as interconnected. This study identified predictors of business performance in a SMEs’ setting. This study presented hypotheses based on four dimensions of CSR. The study shows that CEOs consider economic and legal responsibilities as one dimension. This finding is consistent with some previous studies (Sarkar and Searcy, 2016; Kim et al., 2020) that suggest economic and legal dimensions constitute required business responsibilities. For example, Sarkar and Searcy (2016) argue that economic responsibility is basic, and it cannot be separated from legal responsibility.

This result indicates that the model has more predictive power for companies with long-term tenured CEOs. We compared the *R*^2^ values, which indicate predictive power of the explanatory variables, between the two groups. The *R*^2^ values for companies with long-term tenured CEOs were greater (0.60 for non-financial performance and 0.37 for financial performance) than those for companies with short-term tenured CEOs (0.46 for non-financial performance and 0.25 for financial performance). It is possible that short-term tenured CEOs have a different set of consideration in assessing the company’s business performance. For example, they may consider other variables (e.g., environmental responsibility) not included in this model. Thus, future studies may want to incorporate some other variables that may be helpful for understanding early-stage CEOs.

### Managerial implications

The study’s practical implications are discussed below. The first implication is related to the different predictors of financial performance for the two groups. CEOs with short-term tenure focus on philanthropic responsibility to improve financial performance. This may be because they feel the pressure to differentiate their business from competitors and use philanthropic activities as a differentiator. An alternative explanation is that young CEOs with short-term tenure are inclined to embracing higher levels of CSR based on their value system. On the other hand, CEOs who have been in the position for a long time may view economic/legal dimension as the primary responsibility because they are better equipped to resist external pressures. The finding suggests that CEOs’ perspectives on CSR dimensions shift as they stay longer in the position. The longer the CEOs stay in the position, the more they focus on economic/legal responsibilities. It seems that long-term tenured CEOs recognize the importance of linking CSR activities to the business bottom line, and view economic/legal dimension as the main engine driving business performance. This finding means that early-stage CEOs should be careful not to overlook the importance of economic/legal responsibility in their pursuit of higher levels of CSR (i.e., ethical, philanthropic). TOMS is a great example that shows businesses should balance among different dimensions of CSR. TOMS, a popular shoes company, was built on the buy-one-give-one model in 2006, but later it had to abandon the practice because of the high cost associated with giving away a pair of shoes per sale to poor people.

Both short-term and long-term tenured CEOs view that philanthropic activities affect the company’s financial and non-financial performance. This may be because philanthropic activities enhance the company’s image, which is helpful for generating revenue. As discussed before, early-stage CEOs tend to believe in the power of philanthropic activities more than their counterparts in enhancing financial performance. This finding suggests that they view that philanthropic activities will help with the revenue/profit increase and expand market share. It seems that CEOs in early tenure utilize higher levels of CSR (e.g., philanthropic) as a strategy to achieve a positive image and reputation. However, they should not lose sight of the fact that economic/legal activities are also important for achieving financial and non-financial performance.

The last implication is related to the effect of ethical responsibility on business performance. While short-term tenured CEOs perceive that ethical responsibility influences both financial and non-financial performance, their counterparts do not. This is an interesting finding because CEOs’ perspective on ethical responsibility seems to change over time. It is apparent that CEOs who have a longer tenure feel ethical activities do not have any direct impact on financial or non-financial performance. This may be due to the fact that ethical behaviors are not usually expressed or promoted explicitly to external stakeholders, and, thus, hard to be materialized through financial or non-financial performance.

### Limitations and directions for future research

The study has some limitations. First, this study collected data from SME CEOs in South Korea. CEOs may have different perspectives on CSR depending on cultures and countries, and, thus, this study should be replicated in other cultural contexts. Second, this study investigated the role of CEO tenure as a moderator of a single firm’s CSR activities and has not considered CEO’s influence on its supply chain practices. Future research can study CEO tenure as a moderator of the alignment between CSR activities and the implementation of enabling technologies for its supply chain (Feng et al., 2017). It would be interesting to study how CEO’s tenure moderates the implementation of supply chain green practices—business performance relationship. In the area of mutual funds, previous research reported a positive correlation between sustainable strategies and superior performance compared to traditional investment strategies (Abate et al., 2021). Hence, future research may want to compare the performance of sustainable investments and traditional investments, to investigate if investments in sustainable projects compensate for the loss of profits. Future studies may want to examine other variables, such as personality, leadership style, major field, gender, and compensation. Third, this study used the four dimensions of CSR of Carroll (1991). Future studies may want to consider CSR related to environmentalism. Environmentalism has become an important issue among consumer activists. Fourth, the study’s use of a survey to collect data is another limitation of the study. Because the study used cross-sectional data, it cannot reveal changes between the time periods (e.g., early-stage CEOs and late-stage CEOs). Using a time-series study will be helpful for understanding how CEOs modify their strategies over time. Fifth, it is necessary to look at the CSR of SMEs from a new perspective by applying a different theoretical framework. This study explains the relationship between CSR and business performance by applying echelons theory and stakeholder theory. Going further than that, useful implications can be drawn by empirically analyzing a new conceptual framework by applying other theories (e.g., resource-based view) in the future. Lastly, in future research, it is advisable to construct a conceptual framework that considers a potential role of sustainability and digital transformation. As SMEs play a major role in economic development, in addition to measuring business performance in the current management aspect, it is also necessary to consider the practice of sustainable management (Shashi et al., 2018). To achieve sustainable management of SMEs, the importance of achieving and improving sustainability performance should be emphasized, including concepts such as environmental CSR, environmental performance, and sustainable production processes and products. In addition, there is a sufficient need for research on digital social responsibility of SMEs in the digital transition period. It can be an interesting topic to examine how CSR activities in online spaces such as social media platforms affect customer behavior and business performance.

## Data availability statement

The raw data supporting the conclusions of this article will be made available by the authors, without undue reservation.

## Author contributions

SM-L, JE-B, and YK-L designed the study, collected the data, and contributed to the literature review, manuscript writing, and data analysis. PS contributed to the literature review, manuscript writing, and data analysis. All authors contributed to the article and approved the submitted version.

## Funding

This work was supported by the Ministry of Education of the Republic of Korea and the National Research Foundation of Korea (NRF-2020S1A5B8104093).

## Conflict of interest

The authors declare that the research was conducted in the absence of any commercial or financial relationships that could be construed as a potential conflict of interest.

## Publisher’s note

All claims expressed in this article are solely those of the authors and do not necessarily represent those of their affiliated organizations, or those of the publisher, the editors and the reviewers. Any product that may be evaluated in this article, or claim that may be made by its manufacturer, is not guaranteed or endorsed by the publisher.

## References

[ref1] Abate G. Basile I. Ferrari P. (2021). The level of sustainability and mutual fund performance in Europe: an empirical analysis using ESG ratings. Corp. Soc. Responsib. Environ. Manag. 28, 1446–1455. doi: 10.1002/csr.2175

[ref2] Ahn S.-Y. Park D.-J. (2018). Corporate social responsibility and corporate longevity: the mediating role of social capital and moral legitimacy in Korea. J. Bus. Ethics 150, 117–134. doi: 10.1007/s10551-016-3161-3

[ref3] Bahta D. Yun J. Islam M. R. Bikanyi K. J. (2021). How does CSR enhance the financial performance of SMEs? The mediating role of firm reputation. Econ. Res. 34, 1428–1451. doi: 10.1080/1331677X.2020.1828130

[ref4] Banker R. D. Potter G. Srinivasan D. (2000). An empirical investigation of an incentive plan that includes nonfinancial performance measures. Account. Rev. 75, 65–92. doi: 10.2308/accr.2000.75.1.65

[ref170] Bassyouny H. Abdelfattah T. Tao L. (2020). Beyond narrative disclosure tone: the upper echelons theory perspective. International Review of Financial Analysis, 101499. doi: 10.1016/j.irfa.2020.101499

[ref6] Bhattacharya C. B. Sen S. Korschun D. (2008). Using corporate social responsibility to win the war for talent. MIT Sloan Manag. Rev. 49, 37–44.

[ref7] Bocquet R. Le Bas C. Mothe C. Poussing N. (2019). Strategic CSR for innovation in SMEs: does diversity matter? Long Range Plan. 52:101913. doi: 10.1016/j.lrp.2019.101913

[ref8] Bowen H. (1953). Social Responsibilities of the Businessman. New York: Harper & Row

[ref9] Carpenter M. A. Geletkanycz M. A. Sanders W. G. (2004). Upper echelons research revisited: antecedents, elements, and consequences of top management team composition. J. Manag. 30, 749–778. doi: 10.1016/j.jm.2004.06.001

[ref11] Carroll A. B. (1991). The pyramid of corporate social responsibility: toward the moral management of organizational stakeholders. Bus. Horiz. 34, 39–48. doi: 10.1016/0007-6813(91)90005-G

[ref12] Cheffi W. Malesios C. Abdel-Maksoud A. Abdennadher S. Dey P. (2021). Corporate social responsibility antecedents and practices as a path to enhance organizational performance: the case of small and medium sized enterprises in an emerging economy country. Corp. Soc. Responsib. Environ. Manag. 28, 1647–1663. doi: 10.1002/csr.2135

[ref13] Chen W. T. Zhou G. S. Zhu X. K. (2019). CEO tenure and corporate social responsibility performance. J. Bus. Res. 95, 292–302. doi: 10.1016/j.jbusres.2018.08.018

[ref14] Choongo P. (2017). A longitudinal study of the impact of corporate social responsibility on firm performance in SMEs in Zambia. Sustain. For. 9:1300. doi: 10.3390/su9081300

[ref15] Ciliberti F. Pontrandolfo P. Scozzi B. (2008). Investigating corporate social responsibility in supply chains: a SME perspective. J. Clean. Prod. 16, 1579–1588. doi: 10.1016/j.jclepro.2008.04.016

[ref16] Claver-Cortés E. Marco-Lajara B. Úbeda-García M. García-Lillo F. Rienda-García L. Zaragoza-Sáez P. C.. (2020). Students’ perception of CSR and its influence on business performance. A multiple mediation analysis. Bus. Ethics Eur. Rev. 29, 722–736. doi: 10.1111/beer.12286

[ref17] Costa R. Menichini T. (2013). A multidimensional approach for CSR assessment: the importance of the stakeholder perception. Expert Syst. Appl. 40, 150–161. doi: 10.1016/j.eswa.2012.07.028

[ref19] Donaldson T. Preston L. E. (1995). The stakeholder theory of the corporation: concepts, evidence, and implications. Acad. Manag. Rev. 20, 65–91. doi: 10.2307/258887

[ref21] Feng Y. Zhu Q. Lai K. H. (2017). Corporate social responsibility for supply chain management: a literature review and bibliometric analysis. J. Clean. Prod. 158, 296–307. doi: 10.1016/j.jclepro.2017.05.018

[ref175] Fombrun C. (1996). Reputation: realizing value from the corporate image (Harvard Business School Press, Boston).

[ref22] Fornell C. Larcker D. F. (1981). Evaluating structural equation models with unobservable variables and measurement error. J. Mark. Res. 18, 39–50. doi: 10.1177/002224378101800104

[ref180] Freeman R. E. (1984). Strategic management: a stakeholder approach. Boston: Pitman.

[ref23] Friede G. Busch T. Bassen A. (2015). ESG and financial performance: aggregated evidence from more than 2000 empirical studies. J. Sustain. Fin. Invest. 5, 210–233. doi: 10.1080/20430795.2015.1118917

[ref24] Friedman M. (2007). “The social responsibility of business is to increase its profits” in Corporate Ethics and Corporate Governance. eds. Zimmerli W. C. Holzinger M. Richter K. (Berlin, Heidelberg: Springer), 173–178.

[ref25] Friedman M. Friedman R. (1990). Free to Choose: A Personal Statement. San Diego, CA: A Harvest Book, Harcourt, Inc

[ref26] Graafland J. Van de Ven B. Stoffele N. (2003). Strategies and instruments for organising CSR by small and large businesses in the Netherlands. J. Bus. Ethics 47, 45–60. doi: 10.1023/A:1026240912016

[ref27] Hair J. F. Black W. C. Babin B. J. Anderson R. E. Tatham R. L. (2006). Multivariate Data Analysis (6). Upper Saddle River, NJ: Pearson Prentice Hall

[ref28] Hair J. F. Hult G. T. M. Ringle C. Sarstedt M. (2016). A Primer on Partial Least Squares Structural Equation Modeling (PLS-SEM). Thousand Oaks: Sage Publications

[ref29] Hambrick D. C. Fukutomi G. D. (1991). The seasons of a CEO's tenure. Acad. Manag. Rev. 16, 719–742. doi: 10.2307/258978, PMID: 10115480

[ref30] Hambrick D. C. Mason P. A. (1984). Upper echelons: the organization as a reflection of its top managers. Acad. Manag. Rev. 9, 193–206. doi: 10.2307/258434

[ref31] Han S.-H. Park H.-J. (2018). The effects of franchise CEO's innovation and CSR passion, and customer orientation on perceived service quality, customer affection, and commitment. Kor. J. Franc. Manag. 9, 17–29. doi: 10.21871/KJFM.2018.06.9.2.17

[ref32] Hasan F. (2021). Corporate social responsibility and agency cost: evidence from the UK retail industry. Int. J. Res. Fin. Manag. 4, 105–115. doi: 10.33545/26175754.2021.v4.i2a.114

[ref33] Henderson A. D. Miller D. Hambrick D. C. (2006). How quickly do CEOs become obsolete? Industry dynamism, CEO tenure, and company performance. Strateg. Manag. J. 27, 447–460. doi: 10.1002/smj.524

[ref34] Henseler J. Ringle C. M. Sarstedt M. (2015). A new criterion for assessing discriminant validity in variance-based structural equation modeling. J. Acad. Mark. Sci. 43, 115–135. doi: 10.1007/s11747-014-0403-8

[ref35] Hou M. Liu H. Fan P. Wei Z. (2016). Does CSR practice pay off in east Asian firms? A meta-analytic investigation. Asia Pac. J. Manag. 33, 195–228. doi: 10.1007/s10490-015-9431-2

[ref36] Hur S.-B. Nor Y.-S. Lee D. (2019). The impact of franchisor's economic and philanthropic CSR on franchisees' economic satisfaction, social satisfaction, and loyalty. Kor. J. Franc. Manag. 10, 25–35. doi: 10.21871/KJFM.2019.9.10.3.25

[ref37] Ikram M. Sroufe R. Mohsin M. Solangi Y. A. Shah S. Z. A. Shahzad F. (2019). Does CSR influence firm performance? A longitudinal study of SME sectors of Pakistan. J. Glob. Respons. 11, 27–53. doi: 10.1108/JGR-12-2018-0088

[ref38] Jamali D. Zanhour M. Keshishian T. (2009). Peculiar strengths and relational attributes of SMEs in the context of CSR. J. Bus. Ethics 87, 355–377. doi: 10.1007/s10551-008-9925-7

[ref39] Jenkins H. (2004). Corporate social responsibility and the mining industry: conflicts and constructs. Corp. Soc. Responsib. Environ. Manag. 11, 23–34. doi: 10.1002/csr.50

[ref40] Jenkins H. (2009). A ‘business opportunity’ model of corporate social responsibility for small-and medium-sized enterprises. Bus. Ethics Eur. Rev. 18, 21–36. doi: 10.1111/j.1467-8608.2009.01546.x

[ref41] Juarez L. E. V. (2017). Corporate social responsibility: its effects on SMEs. J. Manag. Sustain. 7, 75–89. doi: 10.5539/jms.v7n3p75

[ref42] Kang T.-W. Sinha P. N. Park C.-I. Lee Y.-K. (2021). Exploring the intra entrepreneurship-employee engagement-creativity linkage and the diverse effects of gender and marital status. Front. Psychol. 12:736914. doi: 10.3389/fpsyg.2021.736914, PMID: 34777125 PMC8578907

[ref43] Khan T. M. Bai G. Fareed Z. Quresh S. Khalid Z. Khan W. A. (2021). CEO tenure, CEO compensation, corporate social and environmental performance in China: the moderating role of coastal and non-coastal areas. Front. Psychol. 11:3815. doi: 10.3389/fpsyg.2020.574062 PMC7862113 33551900

[ref44] Khan T. M. Gang B. Fareed Z. Yasmeen R. (2020). The impact of CEO tenure on corporate social and environmental performance: an emerging country's analysis. Environ. Sci. Pollut. Res. 27, 19314–19326. doi: 10.1007/s11356-020-08468-y, PMID: 32212078

[ref45] Kim B. Lee S. Kang K. H. (2018). The moderating role of CEO narcissism on the relationship between uncertainty avoidance and CSR. Tour. Manag. 67, 203–213. doi: 10.1016/j.tourman.2018.01.018

[ref46] Kim Y. Lee S. S. Roh T. (2020). Taking another look at airline CSR: how required CSR and desired CSR affect customer loyalty in the airline industry. Sustain. For. 12:4281. doi: 10.3390/su12104281

[ref47] Kim J. Song H. Lee C.-K. Lee J. Y. (2017). The impact of four CSR dimensions on a gaming company’s image and customers’ revisit intentions. Int. J. Hosp. Manag. 61, 73–81. doi: 10.1016/j.ijhm.2016.11.005

[ref48] Kock N. (2015). Common method bias in PLS-SEM: a full collinearity assessment approach. Int. J. e-Collab. 11, 1–10. doi: 10.4018/ijec.2015100101

[ref49] Lantos G. P. (2001). The boundaries of strategic corporate social responsibility. J. Consum. Mark. 18, 595–632. doi: 10.1108/07363760110410281

[ref50] Le T. T. (2022). Corporate social responsibility and SMEs' performance: mediating role of corporate image, corporate reputation and customer loyalty. Int. J. Emerg. Mark. doi: 10.1108/IJOEM-07-2021-1164 [Epub ahead of print].

[ref51] Lee M. D. P. (2011). Configuration of external influences: the combined effects of institutions and stakeholders on corporate social responsibility strategies. J. Bus. Ethics 102, 281–298. doi: 10.1007/s10551-011-0814-0

[ref52] Lee K. H. Herold D. M. Yu A. L. (2016). Small and medium enterprises and corporate social responsibility practice: a Swedish perspective. Corp. Soc. Responsib. Environ. Manag. 23, 88–99. doi: 10.1002/csr.1366

[ref53] Lee Y.-K. Lee K. H. Li D.-X. (2012). The impact of CSR on relationship quality and relationship outcomes: a perspective of service employees. Int. J. Hosp. Manag. 31, 745–756. doi: 10.1016/j.ijhm.2011.09.011

[ref54] Li H. Hang Y. Shah S. G. M. Akram A. Ozturk I. (2020). Demonstrating the impact of cognitive CEO on firms’ performance and CSR activity. Front. Psychol. 11:278. doi: 10.3389/fpsyg.2020.00278, PMID: 32184747 PMC7058782

[ref55] Li F. Li T. Minor D. (2016). CEO power, corporate social responsibility, and firm value: a test of agency theory. Int. J. Manag. Financ. 12, 611–628. doi: 10.1108/IJMF-05-2015-0116

[ref56] Lu J. Ren L. Zhang C. Rong D. Ahmed R. R. Streimikis J. (2020). Modified Carroll’s pyramid of corporate social responsibility to enhance organizational performance of SMEs industry. J. Clean. Prod. 271:122456. doi: 10.1016/j.jclepro.2020.122456

[ref57] Magrizos S. Apospori E. Carrigan M. Jones R. (2021). Is CSR the panacea for SMEs? A study of socially responsible SMEs during economic crisis. Eur. Manag. J. 39, 291–303. doi: 10.1016/j.emj.2020.06.002 PMC7398866 38620270

[ref58] Maignan I. Ferrell O. C. Hult G. T. M. (1999). Corporate citizenship: cultural antecedents and business benefits. J. Acad. Mark. Sci. 27, 455–469. doi: 10.1177/0092070399274005

[ref59] Matten D. Moon J. (2008). “Implicit” and “explicit” CSR: a conceptual framework for a comparative understanding of corporate social responsibility. Acad. Manag. Rev. 33, 404–424. doi: 10.5465/amr.2008.31193458

[ref60] McCarthy S. Oliver B. Song S. (2017). Corporate social responsibility and CEO confidence. J. Bank. Financ. 75, 280–291. doi: 10.1016/j.jbankfin.2016.11.024

[ref61] McClelland P. L. Barker V. L. III Oh W.-Y. (2012). CEO career horizon and tenure: future performance implications under different contingencies. J. Bus. Res. 65, 1387–1393. doi: 10.1016/j.jbusres.2011.09.003

[ref62] Meier O. Schier G. (2021). CSR and family CEO: the moderating role of CEO's age. J. Bus. Ethics 174, 595–612. doi: 10.1007/s10551-020-04624-z

[ref63] Miller D. (1991). Stale in the saddle: CEO tenure and the match between organization and environment. Manag. Sci. 37, 34–52. doi: 10.1287/mnsc.37.1.34

[ref64] Miller D. Shamsie J. (2001). Learning across the life cycle: experimentation and performance among the Hollywood studio heads. Strateg. Manag. J. 22, 725–745. doi: 10.1002/smj.171

[ref65] Ministry of SMEs and Startups (2022). Korean SMEs statistics, Available at: https://www.mss.go.kr/site/smba/foffice/ex/statDB/temaList.do (Accessed October 10, 2000)

[ref66] Moneva-Abadía J. M. Gallardo-Vázquez D. Sánchez-Hernández M. I. (2019). Corporate social responsibility as a strategic opportunity for small firms during economic crises. J. Small Bus. Manag. 57, 172–199. doi: 10.1111/jsbm.12450

[ref67] Morgeson F. P. Aguinis H. Waldman D. A. Siegel D. S. (2013). Extending corporate social responsibility research to the human resource management and organizational behavior domains: a look to the future. Pers. Psychol. 66, 805–824. doi: 10.1111/peps.12055

[ref68] Mubeen R. Han D. Abbas J. Álvarez-Otero S. Sial M. S. (2021). The relationship between CEO duality and business firms’ performance: the moderating role of firm size and corporate social responsibility. Front. Psychol. 12:669715. doi: 10.3389/fpsyg.2021.669715, PMID: 35035363 PMC8757377

[ref69] Naseem T. Shahzad F. Asim G. A. Rehman I. U. Nawaz F. (2020). Corporate social responsibility engagement and firm performance in Asia Pacific: the role of enterprise risk management. Corp. Soc. Responsib. Environ. Manag. 27, 501–513. doi: 10.1002/csr.1815

[ref70] Nejati M. Quazi A. Amran A. Ahmad N. H. (2017). Social responsibility and performance: does strategic orientation matter for small businesses? J. Small Bus. Manag. 55, 43–59. doi: 10.1111/jsbm.12305

[ref71] Nguyen P. Rahman N. Zhao R. (2018). CEO characteristics and firm valuation: a quantile regression analysis. J. Manag. Gov. 22, 133–151. doi: 10.1007/s10997-017-9383-7

[ref72] Nirino N. Battisti E. Ferraris A. Dell'Atti S. Briamonte M. F. (2022). How and when corporate social performance reduces firm risk? The moderating role of corporate governance. Corp. Soc. Responsib. Environ. Manag. 29, 1995–2005. doi: 10.1002/csr.2296

[ref73] Oduro S. Bruno L. Maccario G. (2021). Corporate social responsibility (CSR) in SMEs: what we know, what we don’t know, and what we should know. J. Small Bus. Entrep., 1–32. doi: 10.1080/08276331.2021.1951064

[ref74] Oh W.-Y. Chang Y. K. Jung R. (2018). Experience-based human capital or fixed paradigm problem? CEO tenure, contextual influences, and corporate social (ir) responsibility. J. Bus. Res. 90, 325–333. doi: 10.1016/j.jbusres.2018.05.034

[ref75] Onali E. Galiakhmetova R. Molyneux P. Torluccio G. (2016). CEO power, government monitoring, and bank dividends. J. Financ. Intermed. 27, 89–117. doi: 10.1016/j.jfi.2015.08.001

[ref76] Palacios-Manzano M. León-Gomez A. Santos-Jaén J. M. (2021). “Corporate social responsibility as a vehicle for ensuring the survival of construction SMEs: The mediating role of job satisfaction and innovation,” in *IEEE transactions on engineering management*.

[ref77] Pastrana N. A. Sriramesh K. (2014). Corporate social responsibility: perceptions and practices among SMEs in Colombia. Public Relat. Rev. 40, 14–24. doi: 10.1016/j.pubrev.2013.10.002

[ref78] Petrenko O. V. Aime F. Ridge J. Hill A. (2016). Corporate social responsibility or CEO narcissism? CSR motivations and organizational performance. Strateg. Manag. J. 37, 262–279. doi: 10.1002/smj.2348

[ref79] Pivato S. Misani N. Tencati A. (2008). The impact of corporate social responsibility on consumer trust: the case of organic food. Bus. Ethics Eur. Rev. 17, 3–12. doi: 10.1111/j.1467-8608.2008.00515.x

[ref80] Podsakoff P. M. MacKenzie S. B. Lee J.-Y. Podsakoff N. P. (2003). Common method biases in behavioral research: a critical review of the literature and recommended remedies. J. Appl. Psychol. 88, 879–903. doi: 10.1037/0021-9010.88.5.879, PMID: 14516251

[ref81] Podsakoff P. M. MacKenzie S. B. Podsakoff N. P. (2012). Sources of method bias in social science research and recommendations on how to control it. Annu. Rev. Psychol. 63, 539–569. doi: 10.1146/annurev-psych-120710-100452 21838546

[ref82] Porter M. Kramer M. (2006). Strategy and society: the link between competitive advantage and corporate social responsibility. Harv. Bus. Rev. 84, 78–92. 17183795

[ref83] Rehman Z. U. Khan A. Rahman A. (2020). Corporate social responsibility's influence on firm risk and firm performance: the mediating role of firm reputation. Corp. Soc. Responsib. Environ. Manag. 27, 2991–3005. doi: 10.1002/csr.2018

[ref84] Russo A. Perrini F. (2010). Investigating stakeholder theory and social capital: CSR in large firms and SMEs. J. Bus. Ethics 91, 207–221. doi: 10.1007/s10551-009-0079-z

[ref85] Sarkar S. Searcy C. (2016). Zeitgeist or chameleon? A quantitative analysis of CSR definitions. J. Clean. Prod. 135, 1423–1435. doi: 10.1016/j.jclepro.2016.06.157

[ref86] Schlägel C. Sarstedt M. (2016). Assessing the measurement invariance of the four-dimensional cultural intelligence scale across countries: a composite model approach. Eur. Manag. J. 34, 633–649. doi: 10.1016/j.emj.2016.06.002

[ref87] Schwartz M. S. Carroll A. B. (2003). Corporate social responsibility: a three-domain approach. Bus. Ethics Q. 13, 503–530. doi: 10.5840/beq200313435

[ref88] Sethi S. P. (1979). A conceptual framework for environmental analysis of social issues and evaluation of business response patterns. Acad. Manag. Rev. 4, 63–74. doi: 10.2307/257404

[ref89] Shashi S. Cerchione R. Centobelli P. Shabani A. (2018). Sustainability orientation, supply chain integration, and SMEs performance: a causal analysis. Bijdragen 25, 3679–3701. doi: 10.1108/BIJ-08-2017-0236

[ref90] Stoian C. Gilman M. (2017). Corporate social responsibility that “pays”: a strategic approach to CSR for SMEs. J. Small Bus. Manag. 55, 5–31. doi: 10.1111/jsbm.12224

[ref91] Thornton J. C. Byrd J. T. (2013). Social responsibility and the small business. Acad. Entrepreneur. J. 19, 41–75.

[ref92] Torugsa N. A. O’Donohue W. Hecker R. (2013). Proactive CSR: an empirical analysis of the role of its economic, social and environmental dimensions on the association between capabilities and performance. J. Bus. Ethics 115, 383–402. doi: 10.1007/s10551-012-1405-4

[ref93] Tran M. D. Adomako S. (2021). How CEO social capital drives corporate social performance: the roles of stakeholders, and CEO tenure. Corp. Soc. Responsib. Environ. Manag. 28, 819–830. doi: 10.1002/csr.2092

[ref185] Wang G. Holmes R. M. Jr Oh I. S. Zhu W. (2016). Do CEOs matter to firm strategic actions and firm performance? a meta‐analytic investigation based on upper echelons theory. Pers. Psychol. 69, 775–862. doi: 10.1111/peps.12140

[ref94] Wei J. Ouyang Z. Chen H. A. (2018). CEO characteristics and corporate philanthropic giving in an emerging market: the case of China. J. Bus. Res. 87, 1–11. doi: 10.1016/j.jbusres.2018.02.018

[ref95] Wright P. Ferris S. P. (1997). Agency conflict and corporate strategy: the effect of divestment on corporate value. Strateg. Manag. J. 18, 77–83. doi: 10.1002/(SICI)1097-0266(199701)18:1<77::AID-SMJ810>3.0.CO;2-R

[ref96] Wu S. Levitas E. Priem R. L. (2005). CEO tenure and company invention under differing levels of technological dynamism. Acad. Manag. J. 48, 859–873. doi: 10.5465/amj.2005.18803927

